# DNA Methylation and Micro-RNAs: The Most Recent and Relevant Biomarkers in the Early Diagnosis of Hepatocellular Carcinoma

**DOI:** 10.3390/medicina55090607

**Published:** 2019-09-19

**Authors:** Angela Cozma, Adriana Fodor, Romana Vulturar, Adela-Viviana Sitar-Tăut, Olga Hilda Orăşan, Flaviu Mureşan, Cezar Login, Ramona Suharoschi

**Affiliations:** 1 Internal Medicine Department, “Iuliu Haţieganu” University of Medicine and Pharmacy, 400012 Cluj-Napoca, Romania; angelacozma40@gmail.com (A.C.); adelasitar@yahoo.com (A.-V.S.-T.); olgaorasan@yahoo.com (O.H.O.); 2 Department of Diabetes and Metabolic Diseases, “Iuliu Haţieganu” University of Medicine and Pharmacy, 400012 Cluj-Napoca, Romania; 3 Department of Cell Biology, “Iuliu Haţieganu” University of Medicine and Pharmacy, 400012 Cluj-Napoca, Romania; romanavulturar@yahoo.co.uk; 4 Department of Surgery, “Iuliu Haţieganu” University of Medicine and Pharmacy, 400012 Cluj-Napoca, Romania; flaviumuresan@gmail.com; 5 Department of Physiology, “Iuliu Haţieganu” University of Medicine and Pharmacy, 400012 Cluj-Napoca, Romania; 6 Faculty of Food Science and Technology, University of Agricultural Sciences and Veterinary Medicine, 400372 Cluj-Napoca, Romania; ramona.suharoschi@usamvcluj.ro

**Keywords:** biomarkers, hepatocellular carcinoma DNA methylation, micro-RNAs

## Abstract

Hepatocellular carcinoma (HCC) is a frequently encountered cancer type, and its alarming incidence is explained by genetic and epigenetic alterations. Epigenetic changes may represent diagnostic and prognostic biomarkers of HCC. In this review we discussed deoxyribonucleic acid (DNA) hypomethylation, DNA hypermethylation, and aberrant expression of small non-coding ribonucleic acid (RNA), which could be useful new biomarkers in the early diagnosis of HCC. We selected the articles on human subjects published in English over the past two years involving diagnostic markers detected in body fluids, cancer diagnosis made on histopathological exam, and a control group of those with benign liver disease or without liver disease. These biomarkers need further investigation in clinical trials to develop clinical applications for early diagnosis and management of HCC.

## 1. Context

Hepatocarcinoma (HCC) is one of the most common forms of cancers, being the sixth most frequently diagnosed cancer and the fourth leading cause of cancer mortality in 2018 [1].

The alarming incidence of HCC is explained by genetic and epigenetic alterations [2], as well as by the presence of risk factors: hepatitis C virus (HCV), hepatitis B virus (HBV) infection, alcohol consumption, smoking, diabetes, dietary exposure to aflatoxins, obesity, and gut microbiota [3].

Currently, imaging (magnetic resonance imaging or computed tomography) and measurement of serum alpha-fetoprotein (AFP) represent the standard evaluation of suspicious liver nodules. However, AFP sensitivity in early detection of HCC is only 39–65% [4]. Therefore, finding new biomarkers useful for early diagnosis of HCC is a priority.

With the advent of biotechnology, data from multiple forms of “omics”, including genomics, epigenomics, transcriptomics, proteomics, and metabolomics have been used to discover molecular candidates with diagnostic value in HCC. In this article, due to the extensive available data reviewed only HCC biomarkers were derived from epigenomic data.

Epigenetics refers to inherited altered gene expression without an alteration of the DNA sequence itself. Epigenetic alterations, such as DNA hypomethylation or hypermethylation and aberrant expression of micro-RNAs have been studied and associated with HCC [5,6]. Epigenetic changes may represent diagnostic and prognostic biomarkers of HCC. Recent evidence shows that circulating free DNA or RNA molecules released from tumor cells into the body fluids such as blood, urine, and saliva may be used as early biomarkers of HCC.

DNA methylation is a process by which methyl groups are added to CpG dinucleotides. Usually, DNA methylation represses gene expression, and aberrant DNA methylation in cancer cells leads to repression of several genes involved in cell cycle regulation, DNA error repair, and apoptosis [7]. It has been shown that abnormal DNA methylation led to the inactivation of several tumor suppressor genes involved in HCC development [8,9,10,11].

HBV infection has been shown to disturb the methylation of many genes, such as Ras association domain family 1 isoform A (*RASSF1A*), cyclin-dependent kinase inhibitor 2A, glutathione S-transferase pi 1 (*GSTP1*), E-cadherin (*CDH1*), and cyclin-dependent kinase inhibitor 1A (p21 or *WAF1*/*CIP1*), while HCV infection has been shown to affect methylation of adenomatous polyposis coli (*APC*), growth arrest and DNA-damage-inducible beta (*Gadd45β*), suppressor of cytokine signaling 1 (*SOCS*-1), and signal transducer and activator of transcription 1 (*STAT1*) genes, as well as O-6-methylguanine-DNA methyltransferase (*MGMT*) [12,13].

MicroRNAs (miRs, miRNAs) are naturally occurring small non-coding RNA molecules (22–25 nucleotides in size) that control gene expression post-transcriptionally, by either mRNA degradation or by blocking translation initiation. They are involved in many physiological processes, such as proliferation, differentiation and apoptosis [14]. However, microRNA dysregulation is associated with carcinogenesis and tumor progression of several cancer types. They act as oncogenes or as tumor suppressors (e.g. miR-182, miR-150) by controlling key genes involved in carcinogenesis [15]. Several microRNAs have been considered recently as biomarkers for prognosis and treatment of cancers, including HCC. Moreover, due to their release and high stability in serum they have emerged as potential markers for noninvasive diagnosis of early stages of cancer. The most recent developments in this field are presented below.

## 2. Evidence Acquisition

### 2.1. Inclusion Criteria

Articles on human subjects, published in English over the past two years (1 January 2017–31 December 2018) were searched using several keyword combinations as shown in Table 1. Those articles including diagnostic markers detected in body fluids, histopathological confirmation of the tumor, and control group of people without liver disease or with benign liver disease were selected.

### 2.2. Exclusion Criteria

Review articles, meta-analyses, commentaries, conference abstracts, duplicates, studies with incomplete data, articles not in English, and in vitro or in vivo studies (animal models) were excluded.

## 3. Results

Table 2 lists both the hypermethylated and hypomethylated genes involved in hepatocarcinogenesis found in studies published over the past 2 years (2017–2018), along with the circulating free DNA and the reference.

The principal methylated genes are discussed, and the current available data are presented in detail.

*RASSF1A* gene hypermethylation is frequently detected in HCC [7,24,25]. Detection of hypermethylated *RASSF1A* in premalignant hepatic lesions has suggested its implication in early carcinogenesis [26]. Hypermethylation of *RASSF1A* was present in 93% of patients with HCC and 58% of HBV carriers compared to 8% of the healthy control group. In a methylation-specific polymerase chain reaction (PCR-MSP) analysis, *APC* hypermethylation was found in 61.5% of patients with HCC compared to 12.5% of patients with cirrhosis, which suggests a pathogenic role of hypermethylation in HCC [27]. Mohamed et al. showed that serum methylated *RASSF1A* levels have a predictive value of 72.5% for early diagnosis of HCC, particularly in patients at high risk (those with HCV infection), and recommended periodic evaluation using this non-invasive test in patients with HCV infection [28].

Chan et al. found *RASSF1A* gene hypermethylation in 93% of serum samples from patients with HCC and HBV and in 58% of blood samples from patients chronically infected with HBV, indicating that *RASSF1A* might be an early event in the pathogenesis of HCC [26]. Other studies generally reporting unsatisfactory survival in patients with hypermethylated *RASSF1A* levels at the time of diagnosis or one year after tumor resection suggest that *RASSF1A* methylation could be a good prognostic marker [22,24,25]. In contrast, Hui-Chen et al. did not identify methylated RASSF1A gene levels in the plasma of HCC patients in Taiwan, despite the presence of the hypermethylated gene in tumor biopsies [18].

Lu showed that methylation of tumor suppressor genes RASSF1A, COX2, and APC is associated with HCC development [17].

The Cyclooxygenase 2 (COX2) is a prostaglandin synthase involved in synthesis of thromboxane and prostacyclin, in response to growth factors, pro-inflammatory cytokines and carcinogens [29]. High COX2 levels promote carcinogenesis through an alteration of angiogenesis, cell proliferation, and apoptosis. Hypermethylation of *COX2* was reported in gastric cancer, colorectal cancer, and HCC, while its overexpression was also reported in the majority of cancer cases [30]. Hypermethylation of *COX2* was found in 25% of 48 HCC tissues compared to 4.2% of non-cancerous tissues [31]. Um et al. [32] showed that *COX2* methylation was not present in normal liver, cirrhotic liver, and low-grade dysplastic nodules, while in high-grade dysplastic nodules and HCC, *COX2* methylation was present. These findings suggest the potential diagnostic role of methylation markers in HCC. Lu [17] evidenced increased *COX2* methylation in the subgroup of patients diagnosed with HCC and HBV compared to healthy patients and patients with chronic HBV. A significant area under the receiver operating characteristic curve (ROC) value of 0.758 was found for the *COX2* gene. However, since previous studies indicate that HBV promotes *COX2* overexpression through transcription factor recruitment and promoter demethylation [33], further clarification of the role of HBV-regulated *COX2* expression in hepatocarcinogenesis is needed.

Dong et al. [16] reported the methylation of several genes, including *RASSF1A*, *APC*, homeobox A9 (*HOXA9*), *GSTP1*, blood vessel epicardial substance (*BVES*), and tissue inhibitor of metallopeptidase inhibitor 3 (*TIMP3*), in the tumor biopsies of 343 patients with HCC, but only the *RASSF1A*, *HOXA9* and *BVES* gene promoters were hypermethylated in the serum of these patients [16]. Moreover, this study evidenced a higher sensitivity of serum hypermethylation of *RASSF1A* compared to AFP (≥20 ng/L) in HCC diagnosis in patients chronically infected with HBV [16].

Zheng et al. [34] showed that DNA methylation level of 10 CpGs could highly accurately differentiate tumors from normal tissues in patients with HCC, with a sensitivity higher than 86% and a specificity close to 100%. Xu et al. [35] found that methylation of circulating tumor DNA (ctDNA) of another set of 10 CpGs could also differentiate patients with HCC from healthy people with more than 83% sensitivity and 90% specificity. Both CpG sets could be used as biomarkers for HCC diagnosis, but neither of these research teams took into account whether other types of cancer might present similar methylation changes; consequently, these biomarkers cannot be HCC-specific. It is extremely useful to identify and validate biomarkers specific for HCC.

Hypermethylation of *GSTP1* in the promoter region, which encodes glutathione S-transferase P1, was found in about 50% of tumor tissues, including HCC [36,37]. It was demonstrated that aberrant *GSTP1* methylation is associated with progression of HCC and with a more invasive disease [38]. A number of studies proposed the *GSTP1* methylation level as a diagnosis marker of HCC, with a sensitivity of 50–75% and a specificity of 70–91%, which is superior compared to *RASSF1* or *APC* genes [39].

A recent meta-analysis [40] evaluating *GSTP1* methylation in the tissues of 646 subjects with HCC, *APC* methylation in 592 patients with HCC, and *SOCS1* methylation in the tissues of 512 subjects with HCC has shown that hypermethylation of these genes was strongly correlated with HCC risk, and suggested that these epigenetic changes could be valuable diagnostic biomarkers of HCC [40]. Another study investigated the methylation levels of *GSTP1, RASSF1A, APC*, and *SFRP1* genes in the plasma of 72 HCC patients and 37 people with benign liver diseases, and showed that methylation level of *RASSF1A* was correlated with the tumor size; methylation level of *GSTP* was correlated with an increase in serum AFP concentrations, while hypermethylation of *SFRP1* was more frequent in women [24]. Overall, the hypermethylation of all these genes had a sensitivity of 84.7% in detecting HCC [24]. In a study carried out by Wang et al., it was shown that methylation of *GSTP1* plays role in hepatocarcinogenesis, because 50% of patients with HCC and 37.5% of cirrhotic patients had *GSTP1* hypermethylation in serum [8].

*CDKN2A* (also known as cyclin-dependent kinase inhibitor 2A), which encodes the inhibitor of cyclin D-dependent kinases, p16, and SOCS3 (which in turn encodes the suppressor of cytokine signaling 3), is another hypermethylated gene detected in the plasma of HCC patients [23,41].

*TGR5* (G protein-coupled bile acid receptor), a membrane-bound receptor with a key role in regulation of biliary and glucose homeostasis, has been shown to be aberrantly methylated in HCC and might be a diagnostic marker similar to *AFP* in differentiating HCC from chronic viral hepatitis B [33]. Activation of *TGR5* significantly inhibits the migration and proliferation of tumor hepatic cells in vitro, while *TGR5* deficiency intensifies hepatocarcinogenesis induced by chemical substances [42].

Recently, other genes such as *GRASP, AKR1B1, NXPE3, MAP9, SPINT2, RSPH9, ZNF154, STEAP4, FBLN1*, and *VIM* were found to be hypermethylated in HCC [43,44].

In terms of hypomethylated DNA, increased hypomethylated LINE-1 type transposase domain containing 1 (*LINE*-1) levels in serum were associated with HCC progression, aggressiveness, and negative prognosis in HCC patients [45,46,47]. Liu et al. reported the presence of *LINE*-1 hypomethylation in 66.7% of serum samples from HCC patients was associated with positive tumor size, HBV antigen, AFP levels, and poor prognosis [22]. Very importantly, it was found that hypomethylation of *LINE*-1 and hypermethylation of *RASSF1A* promoter were correlated with early recurrence and worse prognosis in HCC patients after tumor resection [22].

Zhang et al. [39] reported the presence of hypermethylation of six genes associated with HCC (*p16, RASSF1A, RUNX3, GSTP1, CDH1*, and *WIF1*), evidencing significant differences between the serum of HCC patients and that of healthy individuals. Furthermore, the same authors [39] showed that the methylation levels of four of the six genes described (*RASSF1A, CDH1, p16*, and *RUNX3*) were significantly different in both tissue and serum samples in patients with HCC. Other authors [17] observed that four genes (*COX2, APC, RASSF1A*, and *miR-203*) presented significantly higher hypermethylation levels in HCC compared to subgroups of subjects without HCC. Interestingly, the hypermethylation levels of the four candidates were considerably increased in HCC subgroups associated with HBV infection (with and without cirrhosis) compared to HCC subgroups associated with HCV infection (with and without cirrhosis), suggesting a high diagnostic value of the four candidates *APC, COX2, RASSF1A*, and *miR-203* in HCC associated with HBV infection. The same authors [17] compared the diagnostic value of the MPM-B model to that of AFP in 113 patients with HCC associated with HBV. They concluded that only 50.44% of the patients had AFP levels over 20 ng/mL, while 84.1% were HCC-positive using the model based on the four genes. These results demonstrated a higher diagnostic accuracy for HCC associated with HBV using the combined model of methylation markers compared to AFP.

Table 3 summarizes the articles published over the past 2 years, presenting the analysis of circulating methylated DNA: sensitivity, specificity, and area under the ROC curve in the early diagnosis of HCC. At the same time, the groups included in the study are mentioned: HCC, cirrhosis, hepatitis B virus infection, hepatitis C virus infection, and healthy adults.

A total of 1126 records were retrieved by a primary PubMed search for microRNAs. After reviewing the titles and abstracts, we selected six microRNA that have proved significant diagnostic value for hepatocellular carcinoma.

MicroRNA-139 was shown to be dysregulated in several tumors, including HCC. Many tumor-suppressive effects of microRNA-139 have been described, including suppression of epithelial–mesenchymal transition and inhibition of proliferation, migration, and invasion [49,50]. Therefore, microRNA-139 was down-regulated in a majority of HCC tissue samples [49], while its low expression was associated with poor prognosis [51]. It has been recently suggested as an early biomarker of HCC. Thus, Li et al. showed that microRNA-139 was reduced in serum of HCC patients as compared with chronic hepatitis B (HBV) control patients, with an AUC of 0.761, specificity of 58.1%, and sensitivity of 80.6% (Table 4) [52]. In another recent study, serum levels of microRNA-139 were very low in HCC patients compared with both chronic hepatitis C patients (HCV) and liver cirrhosis patients, suggesting that microRNA-139 could be an early marker in detection of HCC-induced HCV [53].

MicroRNA-182 has been recently investigated as a candidate biomarker in HCC patients. MicroRNA-182 was investigated by qRT-PCR in the serum of 103 HCC patients, 95 benign liver disease patients (chronic hepatitis, liver cirrhosis and non-alcoholic fatty liver disease (NAFLD)), and 40 healthy people (Table 4) [54]. miR-182 was upregulated in HCC patients compared with benign liver diseases or control subjects, with an AUC of 0.911 between HCC and benign liver disease, with a sensitivity of 78.6% and a specificity of 91.6%.

Quite discordant results have been published recently by Shaheen et al. The serum level of miR-182 was significantly lower in HCC patients as compared with non-cirrhotic HCV patients (non-significant compared with cirrhotic patients). Actually, there was a significant downregulation of miR-182 in cirrhotic HCV patients vs. non-cirrhotic HCV patients. Therefore, miR-182 was a better predictor of fibrosis progression and development of liver cirrhosis than of tumorigenesis [55]. They showed also that microRNA-150 can differentiate HCC patients from the healthy individuals, with a AUC of 0.638, sensitivity of 60%, and specificity of 70% [55].

MicroRNA-331-3p was shown to be affected in different cancer types, including HCC, with promising prognostic value as it was recently shown to induce tumor proliferation and metastasis [54]. Serum microRNA-331-3p was investigated as a potential biomarker for early diagnosis in the same cohort as above for miR-182 (Table 4) [54]. Upregulation of miR-331-3p might discriminate between HCC and benign liver disease with an AUC of 0.890, sensitivity of 79.6%, and specificity of 86.3%.

Altered expression of miR-199a-3p has been described in different cancer types as compared to normal tissue. In HCC, expression of miR-199a-3p is significantly low, while its overexpression remarkably suppresses metastasis, invasion, and angiogenesis in HCC [56]. The potential diagnostic value of lower serum levels of miR-199a-3p was investigated in the serum of 78 HCC patients and in 156 control individuals by qRT-PCR (Table 4) [57]. The ROC curve analysis showed an AUC of 0.883, with 71.8% sensitivity and 86.1% specificity.

In a microRNA-based score, compared with the use of single miRNAs, the combination of several miRNAs seems to have a better diagnostic accuracy. Lin et al. built a serum microRNA classifier (microRNA-29a, microRNA-29c, microRNA-133a, microRNA-143, microRNA-145, microRNA-192, and microRNA-505) that could detect HCC with higher accuracy than α-fetoprotein at a cutoff of 20 ng/mL in 491 participants (healthy individuals, inactive HBV carriers, patients with chronic HBV hepatitis or liver cirrhosis, and patients with HCC) [59]. Compared with α-fetoprotein, the miRNA classifier showed higher sensitivity (79.6% vs 53.8%; *p* <0.0001) and larger AUC (0.833 vs. 0.727, *p* = 0.0018) for both early-stage and small-size HCC (tumor size ≤3 cm) in all participants. Moreover, they tested the classifiers’ ability to predict preclinical HCC in a nested case–control study (27 patients with HCC and 135 matched individuals with HBV hepatitis or liver cirrhosis) that analyzed samples collected 12 months before clinical diagnosis [59].

Using an integrated bioinformatics approach Shi et al. developed a microRNA-based score (microRNA-221, microRNA-21, microRNA-223, microRNA-130a) for HCC detection, with high accuracy (AUC = 0.982) [60].

Zekri et al. investigated the serum expression of 13 microRNAs in 384 patients with HCV-induced chronic liver disease (192 with HCC, 96 with chronic HCV, and 96 with liver cirrhosis) and 96 healthy subjects. They showed that a panel of microRNA-122, microRNA-885-5p, and microRNA-29b provided high diagnostic accuracy (AUC = 0.898) for the early detection of HCC in the normal population, while using a microRNA panel of microRNA-122, microRNA-885-5p, microRNA-221, and microRNA-181b provided good diagnostic accuracy (AUC = 0.845) for early detection of HCC in liver cirrhosis patients [61].

These biomarkers need further investigation in clinical trials in order to develop clinical applications for the early diagnosis and management of HCC.

## Figures and Tables

**Table 1:** Keyword combinations used to search PubMed in the period 01 January 2017–31 December 2018.

‘‘DNA methylation’’ AND ‘‘hepatocellular carcinoma’’	107
“microRNA OR miRNAs” AND ”hepatocellular carcinoma”	1126
”microRNA-139 OR miRNA-139 OR miR-139” AND “hepatocellular carcinoma”	15
”microRNA-182 OR miRNA-182 OR miR-182” AND “hepatocellular carcinoma”	8
”microRNA-331 OR miRNA-331 OR miR-331” AND “hepatocellular carcinoma”	2
“microRNA-199a-3p OR miRNA-199a-3p OR miR-199a-3p” AND “hepatocellular carcinoma”	12
“microRNA-miR-375 OR miRNA-miR-375 OR miR-miR-375” AND “hepatocellular carcinoma”	14
“microRNA-miR-150 OR miRNA-miR-150 OR miR-miR-150” AND “hepatocellular carcinoma”	7

**Table 2:** Summary of published articles retrieved from Pubmed (2017–2018) regarding the role of DNA methylation in non-invasive diagnosis of liver cancer.

DNA Alterations.	Gene	Circulating Free DNA / Number of Cases (%)	Reference
Hypermethylation	*APC*	36/98 (36.7)	[16]
	*APC*	47 /3 57 (13)	[17]
	*BVES*	29/98 (29.6)	[16]
	*COX2*	—	[17]
	*CDKN2A*	50/237 (21.3)	[18]
	*GSTP1*	17/98 (17.3)	[16]
	*HOXA9*	20/98 (20.4)	[16]
	*IGFBP7*	5.33%	[19]
	*P16*	85/119 (71.43)	[20]
	*HCCS1*	75/120 (62.5)	[21]
	*RASSF1A*	51/98 (52.0)	[16]
	*RASSF1A*	77/105 (73.3)	[22]
	*RASSF1A*	21/237 (8.9)	[18]
	*SFRP1*	73/119 (61.34)	[20]
	*SOCS3*	23/48 (47.91)	[23]
	*STEAP4*	30/237 (13)	[18]
	*TBX2*	179/237 (75.5)	[18]
	*TIMP3*	11/98 (11.2)	[16]
	*VIM*	31/237 (13.1)	[18]
	*ZNF154*	135/237 (60.3)	[18]
Hypomethylation	*LINE-1*	80/119 (67.23)	[20]
	*LINE-1*	70/105 (66.7)	[22]

**Table 3:** List of studies reporting the analysis of circulating free DNA in hepatocellular carcinoma (HCC) patients (2017–2018): sensitivity, specificity, and area under the ROC curve.

Study	Number of Patients	Comparison/Control Patients (Number)	DNA Methylation	Se^1^	Sp^2^	AUC	Reference
Dong 2017	343	98 HCC 75 liver cirrhosis 90 chronic hepatitis B 80 healthy individuals	APC	36.7	96.4	0.650	[16]
			RASSF1A	52	91.5	0.718	
			BVES	29.6	97.6	0.636	
			TIMP3	11.2	98.8	0.356	
			GSTP1	17.4	98.7	0.486	
			HOXa9	20.4	95.8	0.521	
			RASSF1A + BVES + HOXa9	83.7	78.9	0.852	
Lu 2017	357	Hepatitis B virus (HBV)-related HCC HBV-related HCC with cirrhosis HCV-related HCC Hepatitis C virus (HCV)-related HCC with cirrhosis HCC without HBV or HCV	APC	-	-	0.644	[17]
			COX2	-	-	0.758	
			RASSF1A	-	-	0.666	
			APC + COX2 + RASSF1A + miR-203	-	-	0.87	
Huang 2018	326	119 HCC 105 liver cirrhosis 52 benign lesion patients 50 healthy people	SFRP1	56.3	26	0.65	[20]
			LINE-1	50.0	8.2	0.70	
			P16	59.4	31.5	0.63	
			SFRP1 + LINE-1 + P16	93.8	63.0	0.86	
Tao 2018	135	80 HBV-related HCC 35 chronic hepatitis B 20 healthy controls	IGFBP7	60.0	77.14	0.695	[19]
Tian 2018	193	20 HCC 146 chronic hepatitis B 27 healthy controls	HCCS1	62.5	83.6	0.730	[21]
			HCCS1+AFP	81.7	52.1	0.713	
Oussalah 2018	289	289 cirrhosis of which 98 had HCC	SEPT9	98	64.4	0.94	[48]
Wei 2017	116	48 HCC 48 non-tumor 10 liver cirrhosis 6 benign lesions 4 normal liver	SOCS3	73.9			[23]
Wu 2017	494	237 HCC 257 control individuals	CDKN29	-	-	-	[18]
			STEAP4	-	-	-	
			ZNF154	-	-	-	
			TBX2	-	-	0.61	
			VIM	-	-	-	
			RASSF1A	-	-	-	

^1^ SE = sensitivity ^2^ SP = specificity.

**Table 4:** Diagnostic value of early microRNA biomarkers for HCC.

	HCC Patients (number)	Comparison/Control Subjects (number)	Plasma	Sensitivity (%)	Specificity (%)	AUC	Reference
miR-139	31	31 HBV hepatitis	Serum	58.61	80.6	0.76	[52]
miR-139	38	42 HCV hepatitis and 45 HCV liver cirrhosis	Serum	85.71	64.29	0.863	[53]
miR-182	103	47 chronic hepatitis 39 liver cirrhosis 9 non-alcoholic fatty liver disease (NAFLD)	Serum	78.6	91.58	0.911	[58]
miR-182	40	20 HCV non-cirrhotic hepatitis	Serum	72.5	65	0.675	[55]
miR-150	40	40 healthy controls	Serum	60	70	0.674	[52]
miR-331-3p	103	47 chronic hepatitis 39 liver cirrhosis 9 NAFLD	Serum	79.61	86.32	0.89	[58]
miR-199a-3p	78	156 healthy controls	Serum	71.8	86.1	0.883	[57]
miR-375	78	156 healthy controls	Serum	52.3	72.7	0.637	[54]
